# Targeting HMGB1: An available Therapeutic Strategy for Breast Cancer Therapy

**DOI:** 10.7150/ijbs.73504

**Published:** 2022-05-09

**Authors:** Haonan Dong, Lu Zhang, Suling Liu

**Affiliations:** Fudan University Shanghai Cancer Center & Institutes of Biomedical Sciences; Cancer Institutes; Key Laboratory of Breast Cancer in Shanghai; The Shanghai Key Laboratory of Medical Epigenetics; Shanghai Key Laboratory of Radiation Oncology; The International Co-laboratory of Medical Epigenetics and Metabolism, Ministry of Science and Technology; Shanghai Medical College; Fudan University, Shanghai 200032, China.

**Keywords:** HMGB1, breast cancer, autophagy, immunogenic cell death, therapy

## Abstract

HMGB1 is a member of highly conserved high mobility group protein superfamily with intracellular and extracellular distribution. Abnormal HMGB1 levels are frequently manifested in various malignant diseases, including breast cancer. Numerous studies have revealed the clinical value of HMGB1 in the diagnosis and therapy of breast cancer. However, the dual function of pro- and anti-tumor makes HMGB1 in cancer progression requires more profound understanding. This review summarizes the functions and mechanisms of HMGB1 on regulating breast cancer, including autophagy, immunogenic cell death, and interaction with the tumor microenvironment. These functions determine the strategies for the development of chemotherapy, radiotherapy, immunotherapy and combination therapies by targeting HMGB1 in breast cancer. Defining the mechanisms of HMGB1 on regulating breast cancer development and progression will facilitate the application of HMGB1 as a therapeutic target for breast cancer.

## Introduction

Breast cancer remains a high incidence worldwide and represents a significant threat to female health 1. Although advances in early diagnosis and therapy, to some degree, have contributed to alleviate the crisis of breast cancer, a large proportion of patients still succumb to a variety of complex malignant phenotypes. The alarming situation urges researchers to find innovative and effective therapeutic targets for breast cancer.

High mobility group protein box 1 (HMGB1) is a member of the high mobility group protein superfamily with secretory and intracellular activity 2, 3. The subcellular localization of HMGB1 determines the manner in which it regulates normal physiological or pathological processes. In the nucleus, HMGB1 acts as a DNA-binding protein, maintaining the stability of chromosomes or interacting with transcription factors to exert co-activator or co-repressor activity through its DNA-binding domains: A-box and B-box 4-6. HMGB1 translocated to the cytoplasm is mainly engaged in Beclin1-mediated autophagy 7. Extracellular HMGB1 is identified as an important damage-associated molecular pattern (DAMP) molecule that interacts with other cells by binding to several receptors, such as RAGE and TLR family members 8-11. Downstream signals activated by HMGB1 are responsible for a variety of biological functions, including autophagy, immunogenic cell death (ICD), release of cytokines and chemokines, angiogenesis and migration 12, 13. Aberrant expression of HMGB1 has been demonstrated and is closely associated with proliferation and metastasis in breast cancer cells 14, 15. More importantly, HMGB1 participates in the regulation of chemo- and radiotherapy resistance in breast cancer 9, 16, 17. Due to the complexity of HMGB1 functions in breast cancer progression, new therapeutic strategies targeting HMGB1 are constantly being developed, such as the identification of potential ICD inducers and the combination therapies with immune checkpoint blockade 18-20.

This review focuses on the function of HMGB1 in breast cancer based on its biological characteristics and summarizes the valuable preclinical and clinical evidence in breast cancer treatment, with the aim of providing insights for the innovation of therapeutic strategies targeting HMGB1.

## The biology of HMGB1

### The distribution of HMGB1

**Figure 1:**
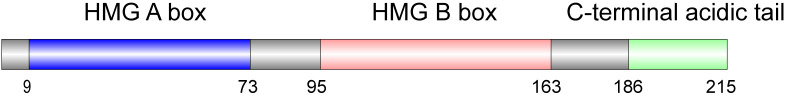
The structure of HMGB1.

HMGB1 is highly expressed in a range of tumor tissues, and its subcellular localization determines the manner in which HMGB1 exerts its regulatory function on cancer. HMGB1 is a 215 amino acid long protein that consists of two DNA-binding domains (HMG A box and HMG B box) and a C-terminal acidic tail (Figure 1). In the nucleus, HMGB1 performs its binding and bending functions with DNA mainly through its A and B box domains respectively 21. The C-terminal acidic tail is composed of continuous aspartate and glutamate residues, and has a protective effect on HMG A and B box during the transport of HMGB1 from the nucleus to the cytoplasm 22, 23. Such strong non-specific DNA affinity of HMGB1 allows its implication in fundamental biological processes such as DNA replication, transcription and modification. Moreover, several transcription factors, such as p53, retinoblastoma (RB) proteins, and NF-κB family members, can enhance their oncogenic activities through direct interaction with HMGB1 6, 15, 24. Furthermore, it has been indicated that loss of HMGB1 activity can increase the frequency of DNA damage. HMGB1 can bind to DNA fragments damaged by chemotherapy or radiotherapy and be engaged in the DNA repair pathway 25. Canonical DNA repair pathways, such as mismatch repair, have been shown to be regulated by nuclear HMGB1 26, 27. Moreover, nuclear HMGB1 alters nucleosome stability in a non-enzymatic, ATP-independent manner 28. There is a partial overlap in the chromatin binding site of HMGB1 and histone H1, which allows HMGB1 to destabilize nucleosome structure by replacing H1 29. Consequently, HMGB1 is critical in maintaining genomic stability. However, more in-depth exploration is required regarding how HMGB1 affects cancer progression through its participation in DNA damage repair. Nuclear HMGB1 is also engaged in the development of serval immune cells. For example, in V(D)J recombination, HMGB1 enhances the activity of RAG1/RAG2 by assembling the RAG-RSS-HMG complex, thereby promotes the rearrangement process of antigen receptor genes, resulting in the diversity of T cell receptors 30, 31. Jiang *et al.* found that HMGB1 silencing could prevent functional skewing of macrophages and downregulate inflammatory factors, suggesting nuclear HMGB1 may serve as a checkpoint molecule of macrophages 32.

In the cytoplasm, HMGB1 is mainly associated with the regulation of autophagy in cancer cells 33. It is necessary to emphasize that acetylation and phosphorylation modifications can prevent HMGB1 from re-localizing in the nucleus, thus facilitating the accumulation of HMGB1 in the cytoplasm. Acetylation of HMGB1 is mediated by the activation of JAK/STAT1 pathway. Specifically, LPS and interferon in the inflammatory microenvironment activate the JAK/STAT1 pathway by binding to TLR4 and initiate HMGB1 acetylation modification 34, 35. Intriguingly, histone deacetylase (HDAC) plays an important role in the deacetylation of HMGB1. For example, SIRT1, a member of class III HDAC, inhibits HMGB1 cytoplasmic translocation through lysine deacetylation 36. Consistent, the treatment of the valproic acid derivative OH-VPA, a histone deacetylase inhibitor, induces the acetylation of HMGB1 and its translocation from the nucleus to the cytoplasm, thus affecting the proliferation of Hela cells 37. Phosphorylation modification promotes HMGB1 localization mainly due to the decreased binding ability of HMGB1 to nuclear import protein KAP-aα1 38. However, the specific molecular mechanism regarding how HMGB1 translocates from the nucleus to the cytoplasm needs to be elucidated in more details.

HMGB1 secretes extracellularly as an immune signal that stimulates the innate immune system to recruit monocytes to inflammatory sites 39. Long-term experimental evidence indicated that extracellular HMGB1 possessed abundant biological activity, such as proliferation, migration, tissue regeneration and anti-bacteria 40. Among them, the most concerned function of extracellular HMGB1 is immune response. HMGB1 induces the release of cytokines and chemokines from immune cells, depending mainly on ligand-receptor interaction. HMGB1 acts as a biomarker of ICD, maturing antigen-presenting dendritic cells and enhancing their antigen-presenting capacity 12. In monocytes and neutrophils, HMGB1 induces the secretion of several pro-inflammatory cytokines, such as IL1-β, TNF-α, and IL-6 41. In addition, extracellular HMGB1 can exert chemotactic effects and support the up-regulation of vascular adhesion molecules, thereby impairing the barrier function of the epithelium 42. Hence, extracellular HMGB1 has a powerful ability to coordinate different immune responses in the tumor microenvironment. Moreover, the level of extracellular HMGB1 has also been proven to correlate with the differentiation of various cell types, such as T cells, stem cells and cancer cells. For example, Li *et al.* have shown that HMGB1 can directly or indirectly promote the differentiation of Th0 cells to Th2 and Th17 cells through RAGE or TLR2 signaling in asthma mouse models 43. In the inflammatory microenvironment, the interaction between HMGB1 and RAGE or TLRs was also implicated in the differentiation of mesenchymal stem cells (MSCs) into vascular endothelial cells and osteoblasts 44, 45. Conversely, the release of HMGB1 may result in the dedifferentiation of cancer cells. For instance, Zhang et al. found that radiation-induced release of HMGB1 promoted the dedifferentiation of CD133^-^ pancreatic cancer cells into cancer stem cell (CSC) phenotype via the TLR2/Hippo/YAP pathway 46. Although targeting HMGB1 may contribute to overcoming metastasis and recurrence of cancer, there are few reports about the association between HMGB1 and CSCs.

### The receptors of HMGB1

The receptor for advanced glycosylation end products (RAGE) is an immunoglobulin superfamily transmembrane receptor that derives its name from the ability to bind to advanced glycosylation end products (AGEs). Due to its inflammatory function in innate immunity, RAGE is commonly considered to be a pattern recognition receptor (PRR) 47. Multiple cellular processes, including proliferation and migration, inflammation and chemotaxis, can be activated via the HMGB1/RAGE signaling axis 48-50. Todorova *et al.* found that HMGB1 interacts with its receptor RAGE in tumor cells but not in normal cells 51. The HMGB1/RAGE signaling axis is also widely involved in breast cancer progression, such as epithelial-mesenchymal transition (EMT), metastasis, and interaction with the tumor microenvironment 52, 53. Interestingly, in addition to the typical characteristics, HMGB1 can also regulate the complications associated with breast cancer. For example, Okui *et al.* found that HMGB1 secreted by breast cancer cells induces bone pain by binding to RAGE of sensory neurons in breast cancer patients with bone metastases 54.

Toll-like receptors (TLRs) are an important group of PRRs participated in non-innate immunity and are also considered to be a bridge between innate and adaptive immunity 55. The function of TLRs in cancer progression has been generally demonstrated 56. Currently, among the TLR family members, TLR2/4/9 were identified as common receptors for HMGB1 57.

As an inflammation-associated receptor, TLR2 is mainly expressed on megakaryocytes and platelets which is related to inflammation-induced vascular disease 58, 59. Notably, Lin *et al.* revealed a non-inflammatory role of the HMGB1/TLR2 axis in breast cancer. They found that in human and mouse breast cancer stem cells, the HMGB1/TLR2 axis promotes NF-κB signaling, production of IL-6 and TGF-β, and activation of STAT3, thereby facilitating self-renewal of CSCs 60.

There are more reports about HMGB1/TLR4 interactions in breast cancer progression. For example, Lv *et al.* reported that macrophage migration inhibitory factors (MIFs) enhance the phosphorylation of ERK and caveolin-1, both of which promote the release of HMGB1 from the nucleus to the cytoplasm and extracellular compartment respectively. Extracellular HMGB1 activates TLR4 signaling and NF-κB phosphorylation, which is responsible for Snail and Twist transcription and MMP2 activation. The HMGB1/TLR4 axis ultimately modifies the intrinsic tumor properties and host immune response in the tumor microenvironment, leading to breast cancer metastasis 61.

Compared to TLR2/4, clarified reports about the interaction of TLR9 with HMGB1 in breast cancer is still absent. CpG DNA or synthetic CpGoligodeoxynucleotide (ODN) analogs can activate TLR9 redistribution from the endoplasmic reticulum (ER) to early endosomes 62. Moreover, CpG ODN also possesses the capacity to stimulate macrophages and dendritic cells (DCs) to secrete HMGB1, which enhances the immunostimulatory potential of CpG ODN in a TLR9-dependent manner 10. In hepatocellular carcinoma (HCC) tumors, HMGB1 mediates tumor growth through interaction with intracellular TLR9 under hypoxic conditions and attracts macrophages to the tumor site, leading to enhanced metastasis 63, 64. However, whether HMGB1 functions as a CpG ODN binding protein in breast cancer is not well known.

### The regulation of HMGB1

As important regulators of post-transcriptional modifications, several miRNAs targeting HMGB1 have been identified, such as miR-107, miR-129-5p, miR-200c and miR-205 52, 65-68. These miRNAs targeting HMGB1 are commonly involved in the regulation of proliferation, migration and therapeutic resistance of breast cancer cells. For instance, the miR-129-5p/HMGB1 axis is implicated in regulating autophagy of breast cancer cells and enhancing their sensitivity to radiotherapy and chemotherapy 66, 67. Particularly, Chen *et al.* found that circRNA CHIPK3 expression was upregulated and predicted a poor prognosis in breast cancer. Mechanistically, cirCHIPK3 serves as a sponge for miR-193a, thereby reactivating HMGB1/PI3K/AKT signaling to promote proliferation, migration and invasion of breast cancer cells 69. Another circRNA, Hsa_circ_0003645 have also been proved that can regulate miR-139-3p/HMGB1 axis to promote breast cancer progression 70. However, there is a paucity of reports on how RNA sponges, or even competing endogenous RNA (ceRNA) regulatory networks, impact HMGB1-mediated breast cancer progression.

Hypoxia is one of the pivotal factors that induce elevated HMGB1 levels in tumor tissues. For example, Huber *et al.* have shown that hypoxia-induced high expression of HMGB1 promotes melanoma growth and metastasis, as well as infiltration of M2 macrophages. In breast cancer, the association between hypoxia and HMGB1 has also been confirmed. For example, HMGB1 can regulate HIF-1α expression in breast cancer cells through the PI3K/AKT signaling pathway 71. Of interest, Sun *et al.* have observed that HMGB1 induces HIF-1α expression while hypoxia may downregulate miR-134-3p, a hypoxia-responsive miRNA, which is involved in the blockade of HMGB1/HIF-1α signaling to inhibit breast cancer progression 72. This finding provides new evidence for the search of potential hypoxia-related targets.

The expression of HMGB1 is regulated by a variety of transcription factors, and the interactions between HMGB1 and transcription factors are complicated. Canonical transcriptional factors, p53 and its homolog p73 exert different effects on HMGB1 promoter activity through binding to another transcriptional factor CTF2: p53/CTF2 downregulates HMGB1 promoter activity, while p73/CTF2 upregulates 73. p53 can not only regulate the expression of HMGB1, but also mediate the release of HMGB1 into the extracellular^74^. Of note, the researchers have demonstrated that HMGB1 can interact with p53 and promote the binding of p53 to DNA through the HMG A box and B box respectively, while the oxidation of the C-terminal acidic tail will impair the binding ability of HMGB1 to p53 75, 76. c-Myc is another transcription factor widely exhibited in cancer cells, and its target genes are involved in many biological processes such as cell cycle, cell adhesion and metabolism. Okuyama* et al.* found that the silence of c-Myc down-regulated the intracellular HMGB1 levels, thus promoting the survival of cancer cells under hypoxia and glucose deficiency 77. The interaction between HMGB1 and transcription factor STAT3 has also been reported in detail. There is one STAT3 binding site on the human HMGB1 promoter 78, and STAT3-mediated upregulation of HMGB1 can also be inhibited by other miRNAs, such as miR-125b and miR-193-3p 79, 80. On the other hand, HMGB1-mediated the activation of STAT3 signal participates widely in tumor progression 81, 82.

Intriguingly, Xu *et al.* demonstrated that HMGB1 can enhance the methylation of STAT3 promoter by recruiting TET2/AID/TDG to the STAT3 promoter region, resulting in the high expression of STAT3 in CD4^+^ T cells, suggesting that HMGB1 may be involved in epigenetic regulation 83.

## The functions of HMGB1 in breast cancer

### Autophagy regulation

Autophagy is an important mechanism for maintaining cellular homeostasis by removing unnecessary or dysregulated cellular components and recycling metabolic substrates. Various autophagic pathways may be regulated to inhibit or promote tumor progression. Multiple stress signals have the capacity to induce autophagy in tumor cells, including metabolic stress and hypoxic stress 84.

**Figure 2:**
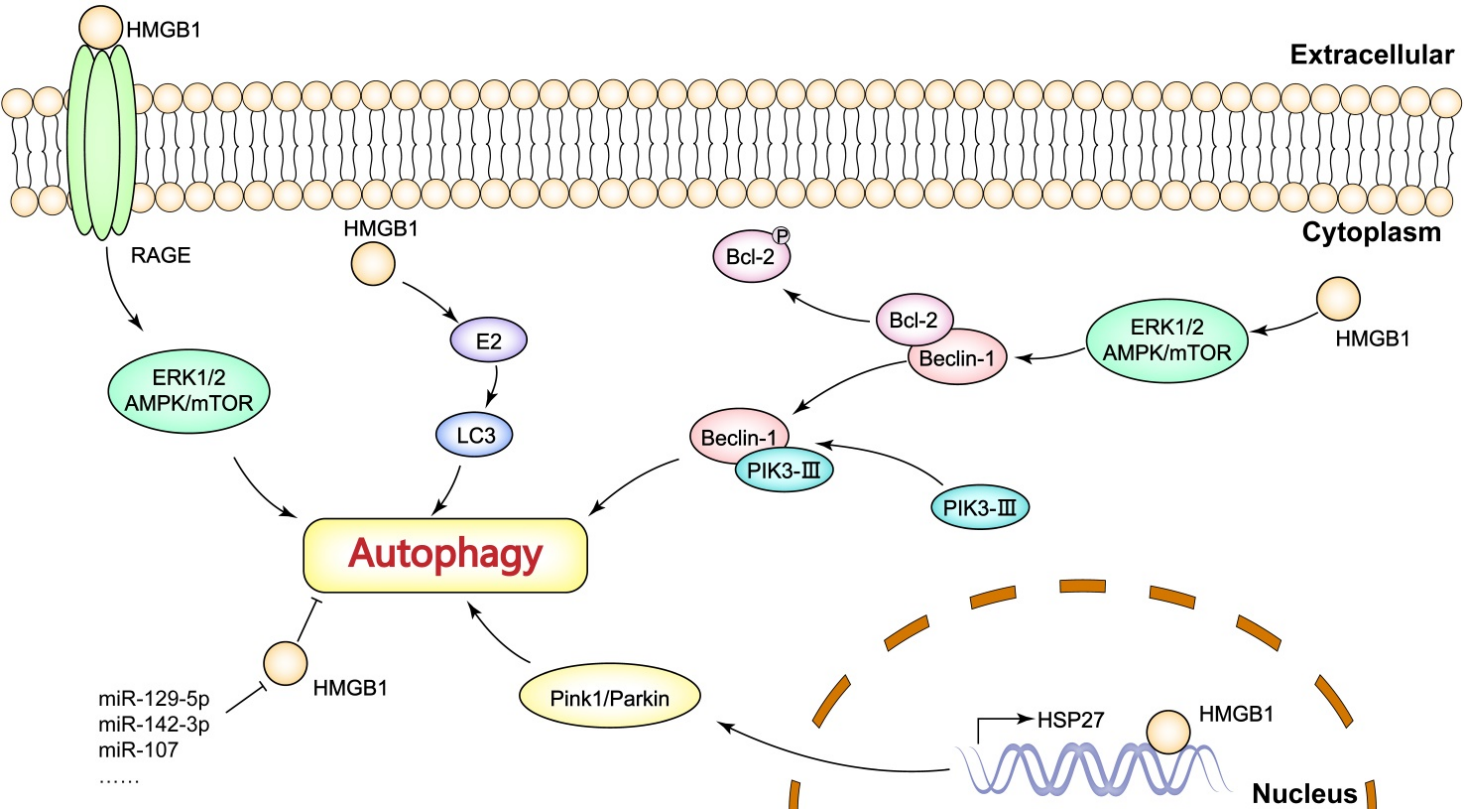
The mechanisms of HMGB1-mediated autophagy in breast cancer.

HMGB1, as an immune signal, is widely recognized as an autophagy inducer. It has been reported that the mechanism of HMGB1-induced autophagy depends on its distribution (Figure 2). In the nucleus, HMGB1 acts as a transcriptional factor based on its DNA binding domains. Tang* et al.* observed that nuclear HMGB1 upregulates the transcriptional level of *HSP27* gene 85. The HMGB1/HSP27 signaling is implicated in the translocation of Parkin and the ubiquitination of VACD1, in which the Parkin/VACD1 pathway plays a critical role in mitophagy 86. These findings suggest that nuclear HMGB1 is largely dependent on the HSP27 pathway to regulate autophagy. In the cytoplasm, HMGB1 promotes Bcl-2 phosphorylation via the ERK/AMPK pathway and induces dissociation of the Beclin1-Bcl-2 complex 87. Critically, dissociation of the Beclin1-Bcl-2 complex promotes the formation of the Beclin1-PI3K-III complex, which is essential in the initiation of cytoplasmic autophagy 7, 88. Especially, extracellular HMGB1 induces the activity of autophagy mainly through receptor-ligand interactions. For example, HMGB1/RAGE signaling can activate Beclin-1-dependent autophagy. Interestingly, Tang *et al.* found that reducible HMGB1 triggers autophagy, while oxidized HMGB1 promotes apoptosis in cancer cells 89. Moreover, ERK1/2 and AMPK/mTOR pathways are also downstream targets of HMGB1/RAGE signaling, and these pathways have been shown to participate in autophagy 90-92.

There are several molecules functioning as key regulators of HMGB1-medaited autophagy in breast cancer. For example, Wang *et al.* validated the role of caveolin-1 and HMGB1 in estradiol (E2)-regulated autophagy 93. Liu *et al.* reported that Med19 inhibition suppresses autophagy by downregulating HMGB1 signaling in breast cancer cells, thus increasing adriamycin chemosensitivity 16. Previous studies have identified several microRNAs that can target HMGB1 to inhibit autophagy in breast cancer cells, such as miR-129-5p, miR-142-3p, miR-107. Particularly, these miRNAs are generally shown to reverse HMGB1-induced resistance to chemotherapy or radiotherapy 65-67, 94, which provide new strategies for breast cancer therapy targeting HMGB1. In addition, HMGB1 is engaged in estradiol (E2)-mediated autophagosome formation in breast cancer. Wang *et al.* found that HMGB1 knockdown down-regulated E2-mediated LC3-II (microtubule-associated protein 1 light chain 3II, an autophagy-associated protein) and Beclin-1 expression as well as inhibited autophagosome formation in BT474 breast cancer cells 93. More importantly, Ladoire* et al.* have reported that nuclear HMGB1 with LC3B (an isoform of LC3) as an independent prognostic marker was significantly associated with prolonged metastasis-free survival 95. In other words, the combined analysis of HMGB1 and autophagy levels is valuable for predicting breast cancer prognosis.

### Immunogenic cell death (ICD) mediation

Immunogenic cell death (ICD) refers to the phenomenon in which tumor cells are transformed from non-immunogenic to immunogenic and mediate anti-tumor immune responses when cancer cells die. ICD involves changes in cell surface components, as well as the release of soluble mediators. These signals, called damage-associated molecular patterns (DAMPs), act on a series of receptors expressed by DCs to stimulate the presentation of tumor antigens to T cells 96. There are various stressors that can induce ICD, such as traditional chemotherapeutic drugs, targeted anti-cancer drugs, and radiotherapy 97.

HMGB1 is one of the DAMPs of ICD, and its release contains two steps involving crossing the nuclear and plasma membrane. HMGB1 that is secreted into extracellular can bind to cell surface receptors on other immune cells, activating downstream signaling pathways and thus enhancing immunity 98. HMGB1, serving as a key biomarker of ICD, has been demonstrated in breast cancer. For instance, HMGB1 can induce ICD through interaction with TLR4, which determines the chemo- or radio-resistance of breast cancer 9. Keita* et al.* have proved that several chemotherapeutic agents significantly triggered HMGB1-involved ICD by immunohistochemistry assessment in breast cancer 99. Additionally, PERK/eIF2α/ATF4/CHOP pathway activates the ER- associated ICD, which induces HMGB1 release 18. These findings suggest that HMGB1-mediated ICD can be a potential target for novel chemotherapy or combination therapy strategies.

### Remodeling the tumor microenvironment

**Figure 3:**
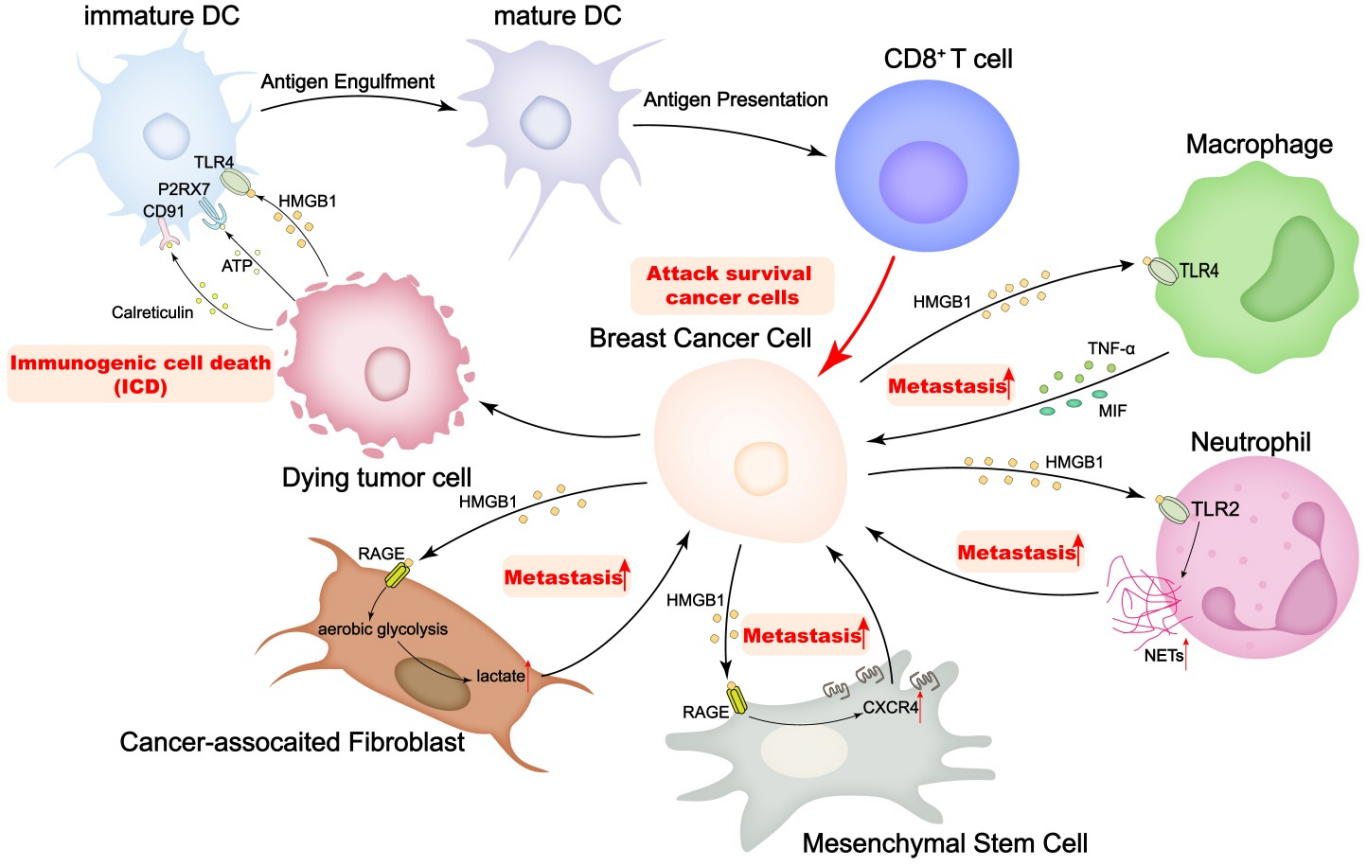
HMGB1 plays important roles on regulating the interactions of breast cancer cells and tumor microenvironment.

Tumor cells, together with various other cells, including immune cells, cancer-associated fibroblasts (CAFs), and MSCs, compose an intricate tumor microenvironment (TME), and the multiplex interactions between different cells in the TME are critical to tumor progression 100. There are numerous experiments showing that HMGB1 executes the function that is widely engaged in dynamic interactions with other cells to remodel the TME, influencing cancer progression (Figure 3). Most impressively, HMGB1 operates as a critical marker of the ICD, contributing to the maturation of DCs and the presentation of antigen to cytotoxic T cells (CTLs), thereby activating CTLs to clear neighboring cells. Aoto *et al.* have found that in breast cancer, HMGB1 interacts with TLR4 on DCs, which is selectively implicated in the initiation of anti-tumor T lymphocytes *in vivo* 9. Release of HMGB1 from dying tumor cells may be required to empower DCs to process and present tumor antigens.

Apart from canonical ICD, interactions of HMGB1 with other components of TME have been reported in recent years. It has been suggested that CD62L^dim^ neutrophils in the pre-metastatic niche are strongly associated with lung metastasis of breast cancer 101. Wang *et al.* have found that, in the mouse TNBC model, the ability of CD62L^dim^ neutrophils to produce neutrophil extracellular traps (NETs) was significantly increased in peripheral blood and lung tissues, which was regulated by tumor-derived HMGB1/TLR2 signaling. Furthermore, the intervention targeting the HMGB1/CD62L^dim^ neutrophil-NETs axis inhibited lung metastasis 102. In addition, HMGB1 promotes TNF-α secretion by macrophages via the TLR-4 pathway 103. Furthermore, MIFs release impacts host immune responses in the TME by regulating the HMGB1/TLR4/NF-κB axis 61, and all these interactions contribute to breast cancer metastasis. Moreover, Hubert *et al.* have observed that HMGB1 inhibition remodels the breast cancer microenvironment, which involves a dramatic reduction in monocyte/granulocyte myeloid-derived suppressor cells and regulatory T lymphocytes, an elevated M1/M2 ratio of tumor-associated macrophages, and an enhancement of DCs and plasmacytoid DCs activation 104. The profound impact of HMGB1 on the TME allows the combination of HMGB1 and other immune cells to be a new target for breast cancer prevention and treatment.

CAFs are the most prominent cell type in the breast cancer microenvironment, and their abundance correlates with high malignancy and poor prognosis. CAFs promote tumor progression by secreting growth factors, enhancing angiogenesis and remodeling the extracellular matrix. Due to their ability to recruit immune cells and influence tumor-immune system interactions, CAFs have been shown to be key players in mediating tumor-promoting inflammation 105. Currently, a bidirectional interaction of HMGB1 between breast cancer cells and CAFs has been partially reported. Amornsupak *et al.* have found that CAFs can induce the production of HMGB1 in breast cancer cells and thus strengthen the chemoresistance to doxorubicin 106. On the other hand, Chen *et al.* have reported that HMGB1 secreted by breast cancer cells can promote aerobic glycolysis and subsequently the activation of fibroblasts through the interaction with RAGE, and then the activated fibroblasts contribute to breast cancer cell metastasis by increasing lactate 53. In addition, tamoxifen elevated HMGB1 expression in CAFs via GPR30/PI3K/AKT signaling, and secreted HMGB1 induced autophagy in an ERK-dependent manner to enhance tamoxifen resistance in MCF-7 cells 107. According to these findings, HMGB1 plays a pivotal role in CAFs-mediated breast cancer progression and chemoresistance, and targeting HMGB1 may be an effective strategy to diminish chemoresistance in breast cancer.

MSCs are early cells of mesodermal development and are widely observed as undifferentiated cells in various tumor tissues 108. A consensus has emerged that MSCs residing in the TME can promote cancer development and progression 109. Ananthula *et al.* have revealed that extracellular acetylated HMGB1 (Ac-HMGB1) can trigger the expression and activation of RAGE in MSCs through *in vivo* and *in vitro* experiments. The activation of RAGE induced CXCR4 expression in MSCs and directed migration to geminin overexpressed (Gem-OE) breast cancer cells. The crosstalk between Gem-OE breast cancer cells and MSCs enhanced the aggressiveness of triple negative breast cancer 110. The notion that geminin overexpression is closely correlated with the proliferation and invasiveness of breast cancer cells, especially triple-negative breast cancer cells, has been widely demonstrated 111-113. Therefore, blocking the geminin-HMGB1/RAGE signaling axis has also been attempted as a strategy to inhibit breast cancer cell proliferation and metastasis 114.

### HMGB1 regulates breast cancer progression

HMGB1 is engaged in many stages of breast cancer progression. However, the dual anti-tumor and pro-tumor biological functions of HMGB1 make its precise role in breast cancer progression quite elusive. The complex role of HMGB1 in cancer cells may depend on the pattern in which HMGB1 is induced. Chemotherapy- and radiation-induced ICDs lead to the release of HMGB1 from dying tumor cells, which usually functions as an immunogenic enhancer 11, 115, 116. While the tumor grows, it may permanently express HMGB1 and undergo special post-transcriptional modifications to promote tumor proliferation, invasion and immune tolerance 117, 118.

The direct effect of HMGB1 on breast cancer cell proliferation is accomplished predominantly through the interaction with retinoblastoma (RB) protein. RB, an oncoprotein, acts a bridge for HMGB1-E2F1 contact by binding to the LXCXE motif of HMGB1. HMGB1 enhances the ability of RB to suppress E2F and cyclin A transcription. This suggests that HMGB1-RB interaction is essential for HMGB1-mediated transcriptional repression, cell growth inhibition, G1 cell cycle arrest, apoptosis induction and tumor growth inhibition 17. The potential association of the LXCXE motif of HMGB1 with RB has been further confirmed. Wang *et al.* constructed point mutants of the LXCXE motif of HMGB1 and found that the interaction of RB with the HMGB1 mutants was disrupted and that HMGB1 significantly inhibited breast cancer cell proliferation through an LXCXE-dependent mechanism 15.

The ability to invade local tissues and subsequently metastasize to distant organs is an important reason for the high mortality rate of breast cancer. Metastasis is a multistep process that includes cell separation from the primary tumor, entry into circulation, extravasation, and colonization in distant organs. The function of HMGB1 in the metastatic process of breast cancer has been more frequently reported. For example, Ni *et al.* have found that HMGB1 silencing inhibited the invasion and migration of human breast cells 14. Mechanistically, HMGB1 can promote angiogenesis and migration of breast cancer through the PI3K/AKT/HIF-1α pathway 71. Another report revealed that the positive function of HMGB1/RAGE signaling pathway in EMT and invasion in triple-negative breast cancer, which the effect can be reversed by miR-205 52. Moreover, hematological and neurological expressed 1-like (HN1L) protein can interact with HSPA9 to upregulate HMGB1 expression and play a key role in promoting breast cancer cell invasion and metastasis 119. Specially, the function of HN1L to promote the properties of CSCs was revealed in breast cancer and prostate cancer 120, 121. Notably, as mentioned above, the interaction of HMGB1 with the TME components, such as neutrophils, macrophages, CAFs and MSCs, also contributes to the metastasis of breast cancer cells.

## HMGB1 serves a potential target breast cancer therapy

### The clinical value of HMGB1 in breast cancer

The clinical value of HMGB1 in breast cancer has been generally established. By immune-histochemical analysis of breast cancer samples, Kostova* et al.* found that HMGB1 in malignant tissues exhibited stronger immuno-reactivity than HMGB1 in normal tissues 8. Sun *et al.* used ELISA to measure serum HMGB1 in the normal population, patients with breast cancer and patients with benign breast disease. They found that the levels of HMGB1 in breast tissues and serum were significantly higher in breast cancer patients than in patients with benign breast disease or the normal population, suggesting that serum HMGB1 may be a useful serological biomarker for the diagnosis of breast cancer 2.

In response to neoadjuvant chemotherapy, Stoetzer* et al.* suggested that HMGB1 and its receptor RAGE could be used to predict and assess breast cancer treatment efficacy 122. Arnold* et al.* found a significant release of HMGB-1 from HCC1143 breast cancer cells treated with epirubicin and docetaxel* in vitro* assay. More importantly, *in vivo* experiments showed that 22 of 41 patients treated with the drug combination who exhibited pathologically complete remission or partial remission responders were accompanied by a significant increase in HMGB1 levels, while the remaining 19 patients did not show any significant changes in breast cancer pathological features with plasma HMGB1 levels 123.

Tumor-infiltrating immune cells are strongly associated with clinical diagnosis and treatment, as well as ideal drug targets to improve cancer patient survival. In breast cancer, Lee* et al.* found that cytoplasmic HMGB1 expression correlated with levels of tumor-infiltrating lymphocytes (TILs) 124. Additionally, the role of extracellular HMGB1 in remodeling the tumor immune microenvironment was also mentioned above. These findings have enriched the understanding of the profound effect of HMGB1 on tumor immune infiltration.

Altogether, the expression pattern of HMGB1 can be applied to the assessment of prognosis, chemotherapy efficacy and immune infiltration of breast cancer, which provides valuable clinical evidence for breast cancer treatment strategies targeting HMGB1.

### HMGB1 and chemotherapy

From different perspectives, HMGB1 enforces different functions in breast cancer chemotherapy. As the most frequently employed approach in breast cancer treatment, the mechanisms in which chemotherapy kills cancer cells depend not only on apoptosis, but also on other non-apoptotic deaths, such as necrosis and autophagy 125. As mentioned above, HMGB1-mediated autophagy occupies an important part in breast cancer. The ability of HMGB1-mediated autophagy to confer chemoresistance of breast cancer cells has been confirmed in various conventional chemotherapeutic drug trials.

There are a variety of miRNAs that can enhance the chemosensitivity of breast cancer cells by targeting HMGB1-mediated autophagy. Shi *et al.* reported that miR-129-5p increased the chemosensitivity of MCF-7 cells to paclitaxel by inhibiting HMGB1-mediated autophagy 67. Liang *et al.* found that miR-142-3p overexpression inhibited autophagy and promoted drug sensitivity to doxorubicin in breast cancer cells through negative regulation of HMGB1 94. The interaction of miRNA with HMGB1 provides new insights into attenuating chemoresistance associated with HMGB1-mediated autophagy.

Moreover, researchers have identified several novel agents that can reverse HMGB1-mediated autophagy-associated chemoresistance. For example, XIAOPI formula was proved to improve breast cancer chemotherapy sensitivity by inhibiting CXCL1/HMGB1-mediated autophagy 126. Interestingly, Zhang *et al.* reported that CDK4/6 inhibitors were found to downregulate HMGB1 expression, inhibit the TLR4/NF-κB pathway, and thereby reverse tamoxifen resistance 127. This suggests the feasibility of HMGB1 as a potential biomarker for screening sensitive patients receiving CDK4/6 inhibitors. Chloroquine, a potent autophagy blocker, has been shown to enhance the chemotherapy sensitivity of cancer 128. In a clinical trial of chloroquine, HMGB1 was applied to assess the level of autophagy of breast ductal carcinoma *in situ* (*clinical trial NCT01023477*) 129. Furthermore, synergistic therapeutic strategies of different drugs targeting HMGB1 have also been reported. Berberine, a clinically approved alkaloid, exhibits stronger antitumor effects in combination with other chemotherapeutic agents 130. More importantly, berberine has been identified as a modulator of HMGB1/TLR4 signaling axis 131, 132. In breast cancer, the synergy of berberine with theophylline induces HMGB1/Bcl-2-mediated apoptosis 133. Zheng *et al.* designed biomimetic co-assembled nanodrug of doxorubicin and berberine, and found that it inhibits tumor growth and pulmonary metastasis by blocking HMGB1/TLR4 signaling, which provides novel evidence to develop safe and efficient nanodrugs for breast cancer 134.

### HMGB1 and radiotherapy

Radiation therapy is a traditional treatment for many malignancies, including breast cancer, and can significantly improve patient survival, despite the damage to surrounding normal tissues. However, there are conflicting reports on the resistance of HMGB1 to breast cancer radiotherapy.

The ability of HMGB1 to enhance the radiosensitivity of breast cancer cells is generally considered to be largely correlated to its immunogenic effects. Apetoh *et al.* found that ionizing radiation promotes the release of HMGB1 from dying tumor cells. HMGB1 activates the TLR4/MyD88 signaling pathway in DCs and thus efficiently processes antigens associated with the presentation of dying tumor cells 9. In addition, the radiation-induced abscopal effect (RIAE) is a miraculous phenomenon that refers to the shrinkage of non-irradiated lesions in contrast to the irradiated tumor lesions. It is now well acknowledged that RIAE relates to the systemic antitumor immune response that is induced by two simultaneous changes in the treatment of tumors: the release of tumor antigens and the exposure of DAMPs 135. As mentioned above, the reaction that produce these changes are associated with ICD. Zhu *et al.* reported that HMGB1 released from radiated breast cancer cells could promote TNF-α secretion by surrounding macrophages through the TLR4 pathway, thus contributing to promote the radiation-induced anti-tumor abscopal effect triggered by breast cancer radiotherapy 103. Although, the exact mechanism of HMGB1 in RIAE has not been adequately elucidated, the role of HMGB1 in bridging the gap between ICD and RIAE remains the importance for the development of immunotherapy in combination with radiotherapy.

Nevertheless, several experiments have shown a positive correlation between HMGB1 and radiotherapy resistance in breast cancer. For example, Luo *et al.* found that HMGB1-mediated autophagy enhanced radiotherapy resistance in breast cancer cells, and this effect was reversible for miR-129-5p 66. Additionally, HMGB1 has also been proven to function in replenishing telomeric DNA. Ke e*t al.* demonstrated that HMGB1 knockdown decreased the levels of telomere-binding proteins such as TPP1, TRF1 and TRF2 as well as inhibited DNA damage repair pathways after shRNA-mediated downregulation of HMGB1 136. In short, downregulation of HMGB1 disrupts telomere homeostasis, inhibits DNA damage repair and enhances radiosensitivity in human breast cancer cells. However, there are only a few studies on the HMGB1-related DNA damage repair pathway as a therapeutic target, so more investigation is required on HMGB1 knockdown as a potential therapeutic option for radiation therapy.

### HMGB1, immunotherapy and combination therapy

Immunotherapy is a therapeutic method to specifically remove small residual tumor lesions, inhibit tumor growth and break immune tolerance by activating immune cells and enhancing the anti-tumor immune response in the body. Based on the powerful immune-modulatory function of HMGB1, immune or combination therapies developed for HMGB1 are of great significance to improve the prognosis and prolong the survival of cancer patients.

Among the currently available HMGB1-related combination therapy strategies, the most frequent is the employment of ICD inducers to activate adaptive anti-tumor immune responses in patients 97. Conventional antitumor chemotherapeutic agents usually are highly cytotoxic and less targetable. The cell death induced by these chemotherapeutic agents tends to be non-immunogenic, and the dying tumor cells can be cleared without activating the immune response, a tolerogenic process that fails to produce a long-term antitumor effect 137. In clinical trials, chemotherapeutic agents such as anthracyclines and oxaliplatin have been observed to induce tumor cell death and confer immunogenicity to the dead tumor cells, activating the immune system to fight these dying tumor cell antigens 138, 139. In breast cancer, researchers have also been identifying new ICD inducers. For instance, oleandrin, a cardiac glycoside, can induce ER stress in breast cancer cells and trigger ICD-mediated immune responses via the PERK/elF2α/ATF4/CHOP pathway 18. In addition, Somma *et al.* found that G-quadruplex binders induced ICD in MDA-MB-231 breast cancer cells, accompanied by T-cell activation 140. Furthermore, Huang *et al.* indicated that the antioxidant N-acetylcysteine (NAC) enhanced not only the antitumor activity of polyglycolylated arginine deiminase (ADI-PEG20), but also the features of ICD, including HMGB1 and ATP secretion 19. Nevertheless, the ICD inducers in clinical trials for breast cancer are limited so far. Researchers showed that P2Et, an extract of Caesalpinia Spinosa, induces the release of HMGB1 and has synergistic effects with anthracycline-type chemotherapeutic agents in animal models of breast cancer. Importantly, the results of a phase Ⅰ clinical study in healthy volunteers suggest that the extract is safe, while other factors, such as the optimal dose of P2Et, are still being explored (*clinical trial NCT05007444*) 141.

Similar to chemotherapeutic agents, oncolytic virus (OV), an *in situ* tumor vaccine, can generate a pro-inflammatory response by producing pathogen-associated molecular patterns (PAMPs) and releasing tumor-associated antigens (TAAs) 142.Gebremeskel *et al.* found that lysovirus-induced tumor cell death exhibited significant features of the ICD including elevated HMGB1 levels. More meaningfully, lysovirus and NKT cell immunotherapy can be effectively combined to reduce tumor load and improve survival in mice with metastatic breast cancer 143. However, while OVs can be significantly improved as powerful cancer treatment candidates, alleviating the limitations of OVs with a combination strategy remains a challenge.

Immune checkpoints refer to the inhibitory signaling pathways that exist in the immune system. Under normal circumstances, the balance of co-stimulatory and co-inhibitory signaling permits the body to maintain immune tolerance. When the body is threatened by tumor burden, the immune checkpoint signaling pathway is generally obstructed, thereby dampening autoimmunity and providing an opportunity for tumor growth and escape 144. Immune checkpoint blockade disrupts the co-inhibitory signaling pathway and activates the anti-tumor function of T cells, while ICD can enhance the T-cell response to tumors. Therefore, establishing a link between ICD and immune checkpoint blockade is a feasible strategy that promises to break through the inherent restrictions of immunotherapy. In recent years, the dual coordination of ICD and immune checkpoint function of several agents has been gradually revealed 145. Li *et al.* discovered that breast cancer cells treated with SR-4835, a CDK12/13 specific inhibitor, exhibited features of ICD, including the release of HMGB1. Critically, enhanced infiltrative activation of DCs and T cells was observed in tumors treated with the combination of SR-4835 and anti-PD-1 antibodies, suggesting that this combination therapy promoted an improved immune response 20. Shan *et al.* indicated that pingyangmycin, an ICD inducer, may enhance the therapeutic efficacy of anti-PD-1 antibodies associated with enhanced tumor infiltrating CD8^+^ T cells, in a similar fashion to SR-4835^146^. However, Hubert *et al.* showed that blocking extracellular HMGB1 could improve the efficacy of anti-PD-1 immunotherapy 104. Besides canonical PD-1/PD-L1 blockade, the potential role of HMGB1 involvement in other immune checkpoint blockade has also been reported. For example, the inhibitory receptor TIM-3 limits antitumor immunity in breast cancer by inhibiting CXCL9 production by XCR1^+^ classical dendritic cells. Pulido *et al.* found that type I interferon and extracellular DNA are required for enhanced CXCL9 expression by splenic cDC1s after TIM-3 blockade, in which DNA uptake is dependent on the DNA-binding ability of HMGB1. TIM-3 limits the activation of the cGAS-STING pathway in intra-tumoral DCs by inhibiting extracellular DNA uptake 147. Undoubtedly, this finding affords a novel perspective for understanding the mechanisms of TIM-3-related immunotherapy.

Tumor vaccines carry tumor-specific antigens (TSAs) or TAAs, which attack tumor cells by stimulating adaptive immunity, overcome the immunosuppressive state caused by tumor products, and improve autoimmunity to destroy tumors 148. Yan *et al.* infected 4T1 cells with recombinant adenovirus AdVEGFR2 carrying the *VEGFR2* gene and found that tumor growth and lung metastasis were inhibited in the vaccinated mice compared to the non-vaccinated mice. *In vitro*, the expression of HMGB1 and HSP70 was increased in 4T1 cells infected with AdVEGFR2, which was responsible for the activation of tumor antigen-specific T cell immunity 149. Table 1 summarizes breast cancer therapy strategies targeting HMGB1 and their effects.

## Conclusions

The pro- and anti-tumor activity of HMGB1 have been reported on its carcinogenicity in breast cancer. The different patterns of HMGB1 induction determine its subcellular distribution, influence HMGB1-related biological processes such as autophagy, immunogenic cell death, and ultimately the efficacy of chemotherapy and radiotherapy for breast cancer. Furthermore, HMGB1-mediated immune activity indicates the new potential about immunotherapy for breast cancer, liberating the possibilities for innovation of combination therapeutic strategies. Although HMGB1 is a high potential target for early detection or treatment of cancers, its dual function in breast cancer remains to be elucidated in more detail.

## Figures and Tables

**Table 1:** Breast cancer therapeutic strategies targeting HMGB1

Therapeutic Strategies	Mechanism	Effect on breast cancer	Ref.
**Chemicals**			
XIAOPI formula	Inhibit CXCL1/HMGB1 signal	Enhance taxol chemosensitivity	126
CDK4/6 inhibitor abemaciclib	Inhibit HMGB1/TLR4/NF-κB pathway	Enahnce tamoxifen chemosensitivity	127
Triptolide	Inhibit HMGB1/TLR4/NF-κB pathway	Suppress breast cancer growth	150
Irinotecan	Increase HMGB1 expression	Induce apoptosis; Arrest cell cycle in S phase	151
**ICD inducers**			
oleandrin	Induce HMGB1 release	Promote DCs maturation and activation; Enhance CD8^+^ T cells cytotoxicity	18
CDK12/13 inhibitor SR-4835	Induce HMGB1 release	Enhance anti-PD-1 anti-cancer activity	20
pingyangmycin	Induce HMGB1 release	Enhance anti-PD-1 anti-cancer activity; Augment tumor-infiltrating CD8^+^ T cells	146
**Synergetic strategy**			
Doxorubicin and berberine	Inhibit HMGB1/TLR4 pathway	Inhibit tumor growth and pulmonary metastasis	134
Theophylline and berberine	Decrease HMGB1 expression	Enhance apoptotic cell death	133
LiCl and MMC	Decrease HMGB1 expression	Enhance MMC-induced apoptosis	152
ADI-PEG20 and NAC	Induce HMGB1 release	Enhance NAC-induced apoptosis	19
Apicidin and docetaxel	Induce HMGB1 release	Enhance apoptotic cell death	153
ALA and Radiotherapy	Increase HMGB1 expression	Enhance RT-induced cellular senescence	154
**Other**			
Recombinant oncolytic VSV	Induce HMGB1 release	Decrease metastatic breast tumor burden	143
Recombinant adenovirus AdVEGFR2	Increase HMGB1 expression	Active tumor antigen-specific T cell immunity; Inhibit angiogenesis, tumor growth and pulmonary metastasis	149

LiCl: lithium chloride; MMC: mitomycin C; ADI-PEG20: pegylated arginine deiminase; NAC: N-acetylcysteine; ALA: alpha-lipoic acid; RT: radiotherapy; VSV: vesicular stomatitis virus.
